# Foudroyant Course of an Extensive *Clostridium septicum* Gas Gangrene in a Diabetic Patient with Occult Carcinoma of the Colon

**DOI:** 10.1155/2013/216382

**Published:** 2013-06-20

**Authors:** Maximilian Hartel, Asad Kutup, Axel Gehl, Jozef Zustin, Lars G. Grossterlinden, Johannes M. Rueger, Wolfgang Lehmann

**Affiliations:** ^1^Department of Trauma, Hand and Reconstructive Surgery, University Medical Center Hamburg-Eppendorf, Martinistraße 52, 20246 Hamburg, Germany; ^2^Department of General, Visceral and Thoracic Surgery, University Medical Center Hamburg-Eppendorf, MartiniStraße 52, 20246 Hamburg, Germany; ^3^Department of Legal Medicine, University Medical Center Hamburg-Eppendorf, Butenfeld 34, 22529 Hamburg, Germany; ^4^Institute of Pathology, University Medical Center Hamburg-Eppendorf, MartiniStraße 52, 20246 Hamburg, Germany

## Abstract

*Background*. Spontaneous gas gangrene is a rare disease in which *Clostridium septicum* frequently can be detected. After an incubation period of 5–48 hours, a very painful swelling is accompanied by a rapidly spreading toxic-infectious clinical picture ultimately leading to septic shock and multiple organ failure. We present a case of a completely documented rare infectious disease with triage findings including initial vital signs, initial medical findings, and the emergency lab., radiological, intraoperative, histopathological, microbiological, and postmortem results. After initial diagnosis of the underlying disease, the patient has been immediately transferred to the operating theatre. The laboratory findings reflect the devastating effect of toxin **α** which is a toxin typically produced by *C. septicum.* The patient presented both an anaemia and a manifest coagulopathy as well as an onset of multiple organ failure. Despite the aggressive medical and surgical measures that have been taken, this patient could not be saved. *Discussion*. The case presented vividly emphasises the difficulty to identify these cases early enough to save a patient. This documentation may help health care providers to identify this life threatening disease as early as possible in future cases.

## 1. Introduction

With the introduction of antibiotics and surgical procedures under sterile conditions, gas gangrenes have become a rare clinical condition [1, 2]. In Germany, for instance, in the year 2000, only 66 cases were recorded [3]. A high percentage of these cases are caused by clostridial strains [1]. Most of the infections (70%) occur posttraumatically or postoperatively. In 80% of these cases, *Clostridium perfringens* can be detected. Other possible strains are *Clostridium haemolyticum*, *Clostridium oedematiens*, *Clostridium novyi*, *Clostridium histolyticum*, or *Clostridium septicum* [2, 4]. In the even scarcer cases of nontraumatic gas gangrenes, *Clostridium septicum* is typically the culprit pathogen [5]. 

## 2. Case Report

A 73-year-old Caucasian male suffered from pain in his right leg and lower lumbar spine without neurological deficit since the same day's morning. Due to an insulin-dependent diabetes, he usually applied his splash into his right thigh. In the afternoon he already went to see a general practitioner, who reportedly prescribed some unspecified painkillers. Due to increasing leg and back pain, he called himself an ambulance and came to our hospital.

At admission, the triage nurse on duty noted the following parameters:

- pain on the visual analogue scale: 4 of 10;
- blood pressure: 125/59 mmHg;
- pulse: 74/minute;
- temperature: 36.6°C;
- respiratory rate: 12/minute;
- oxygen saturation: 97% in ambient air.

The triage nurse admitted the patient with a suspected diagnosis of sciatica. 

Forty-five minutes after the patient first contacted the triage nurse, he described markedly increasing pain levels. Therefore, he was promptly seen by a resident specialized in orthopaedic trauma surgery. The patient stated to be an insulin-dependent type II diabetic. Furthermore, he reported unintended weight loss in the shorter history. At that time, the patient still presented normal vital signs. 

**Figure 1:**
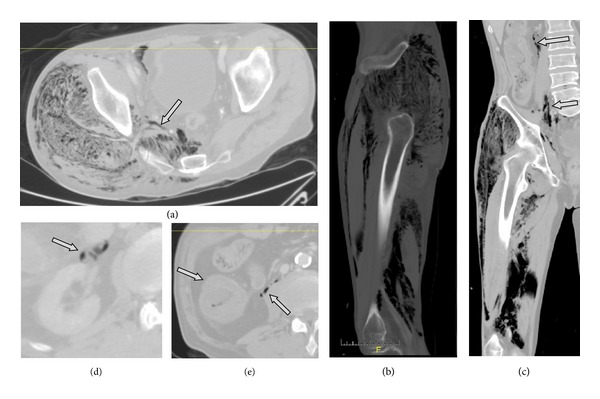
Preoperative CT scan. (a) shows an axial slice of the pelvis a little proximally from the acetabula with a massive infestation of the gluteal muscles and the intrusion of the infection into the retroperitoneal space in the true pelvis though the sciatic notch (arrow). (b) shows a sagittal reconstruction slice with extensive infestation of the whole circumference of the thigh. Gluteal musculature, the extensor muscles (quadriceps and rectus), and hamstrings are affected. (c) shows a coronary reconstruction with again massive affection of the thigh. It also indicates the disseminated infestation of the retroperitoneum with evidence of gas (arrows). (d) shows gas next to the vascular pedicle of the right kidney (arrow). (e) shows the thickened ascending colon (left arrow) and again retroperitoneal gas (right arrow).

The clinical examination showed an increased circumference of the right thigh. Macroscopically, the integument was intact and normally perfused. With a maximum above the fascia lata, a circumferential soft tissue emphysema of the right thigh was noted, extending distally to the lower leg and proximally the right sacral area. However, the overall clinical situation at this time was fairly unremarkable and could only be perceived with a thorough examination from the outside. The patient was promptly treated with crystalloid fluid, ampicillin/sulbactam, and piritramide (opioid analgesic derivate, very commonly used in central Europe). The first venous blood gas analysis indicated the severity of the sepsis with a pH of 7.10, a base excess of −17.4 and a lactate of 10. With the strong suspicion of gas gangrene, the patient—at that point still cardiopulmonary stable—was prepared to be immediately transferred to the operation theatre. The medical team consisting of orthopaedic trauma surgeons and general surgeons decided to perform an explorative CT scan on the way to the operation theatre, in order to assess the extent of infestation. It showed the affection of almost the entire right lower extremity and, worse, the intrusion into the retroperitoneal space ranging up to the vascular pedicle of the right kidney. Furthermore, the CT scan revealed a long-distance thickening of the ascending colon allowing a chronic inflammation or a neoplasm as a differential diagnosis. Finally, the scan showed a chronic calcifying pancreatitis Figure 1. 

Forty minutes after the first medical contact, the patient reached the operation theatre. 

The following exploratory emergency operation, performed by a team of general and orthopedic trauma surgeons, confirmed the clinical diagnosis of gas gangrene. Due to the extensive intraoperative findings after opening of the fascia, we discussed the amputation of the right leg by performing a hip exarticulation. 

In spite of generous fluid administration, blood transfusion, and correction of coagulopathy (tranexamic acid, prothrombin complex concentrate, plasma, and platelets), a markedly increasing catecholamine dependency was noted within seconds after beginning of the operation. Finally, stable conditions could not be obtained any more under excessive catecholamine administration. An interdisciplinary team consisting of anaesthetists, orthopaedic trauma surgeons, general surgeons, and intensive care physicians decided to discontinue the therapeutic measures due to an infaust prognosis. The patient died just less than 3 hours after the initial medical consultation. 

### 2.1. Laboratory Findings

During surgery, several samples from the retroperitoneal space, the thigh, and lower leg were sent out for pathologic and microbiologic workup. 

**Figure 2:**
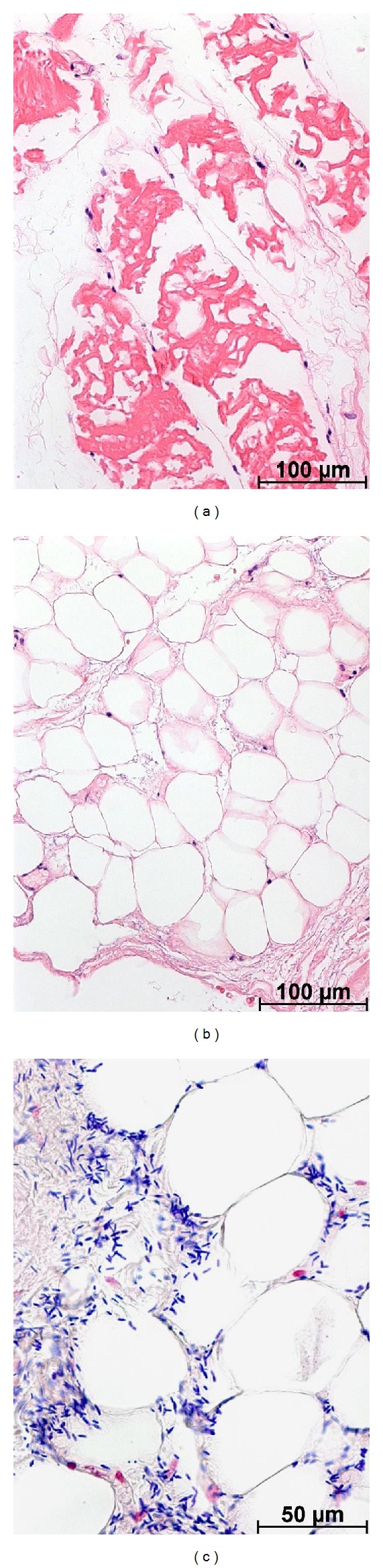
Histopathological findings. (a) Muscle fibers showed irregular condensation of sarcoplasm with no stainable nuclei of muscle cells. Several fibroblasts were apparent between the necrotic muscle fibers (stain: hematoxylin-eosin, original magnification: ×200). (b) Adipocytes and fibrous tissue displayed edema and only few stainable nuclei, consistent with soft tissue necrosis (hematoxylin-eosin: ×200). (c) Between the necrotic cells, numerous large, blunt-ended rods were apparent, consistent with *Clostridium* bacteria (Gram stain: ×400).

The tissue designated for the pathology department was immediately fixed in 4% buffered formalin. The soft tissue was embedded in paraffin and stained with hematoxylin-eosin and Gram stains. Microscopically, the soft tissue biopsy displayed loss of stainable nuclei in both muscle (see Figure 2(a)) and fat tissue (Figure 2(b)). Numerous large Gram-positive blunt-ended rods were apparent throughout the necrotic soft tissue in sections stained with Gram stain. (Figure 2(c)). 

A strain of *Clostridium septicum* was then cultivated in the microbiological samples. 

See Table 1 with a selection of the emergency blood test results. 

### 2.2. Postmortem Findings

Macroscopically, the examiners noted a toxico-infectious shock as primary cause of death. As anticipated in the in vivo CT scan, an extensive manifestation of gas gangrene of the entire right lower limb could be found, continuing into the entire retroperitoneal space. A generalised arteriosclerosis grade II-III (0–IV) was found as well as a three-vessel coronary heart disease and a moderately enlarged left ventricle. A circular stenosing mucinous colon ascendens carcinoma (6 × 1 cm) with infiltration of the pericolic fatty tissue was found without distant or lymph node metastases. Further findings were an obstructive lung disease and a higher grade osteoporosis. 

## 3. Discussion

Similar cases have been reported in the literature [6–10]. However, the case presented in this paper is, to our knowledge, uniquely well documented. 

*Clostridium septicum*, a comparably aerotolerant strain, was the first anaerobic germ to be cultured by Louis Pasteur in 1877 [11, 12]. It is a Gram-positive, motile, and spore-building rod. The motility of *C. septicum* is thought to be a significant virulence factor in clinical disease [13]. Like *Clostridium perfringens*, it can be found in the normal gut flora in humans [14]. After an incubation period of 5–48 hours, a possibly very painful swelling is accompanied by a rapidly spreading toxic-infectious clinical picture, ultimately leading to septic shock and multiple organ failure [1, 13, 15]. Next to several toxins and enzymes build by *C. septicum*, the toxin *α* causing hemolysis is mainly held responsible for the severity of the disease [16]. The blood test results of the case presented reflect the devastating effect of toxin *α*. The patient presented both an anaemia and a manifest coagulopathy as well as an onset of multiple organ failure (Table 1). If left untreated, manifest infections with *Clostridium septicum* almost always lead to fatal outcomes. Early and aggressive medical and surgical intervention is of utmost importance [16]. After finding the right diagnosis, the treatment team managed to quickly transfer the patient to the operating theatre. Despite the aggressive medical and surgical measures taken, this patient could not be saved. The mortality for spontaneous *C. septicum* gas gangrene is estimated to be as high as 65%. Most deaths occur within 24–48 hours after onset of the disease [13]. The case presented vividly emphasises the difficulty to identify these cases early enough to save a patient.

Fatal infections with gas-forming bacteria are most commonly found after prior trauma with application of perforating force (e.g., MVAs, gunshot wounds, agricultural-related trauma, illegal abortions, or after surgical treatments). The latter has become rare after introduction of preoperative antibiotic prophylaxis [1, 17]. Cases with gas gangrene localized at sites of damaged soft tissue are most commonly linked to infections with *Clostridium perfringens*. 

Nontraumatic, endogenous gas gangrene is primarily found in the elderly [10, 17]. Infections with *Clostridium septicum* have especially been linked to malignancy of the bowel, leukaemia, diabetes mellitus, and drug-induced immunosuppression [10, 12]. Larson et al. found in their case series that all patients suffering of spontaneous gas gangrene had either malignancy or some form of immunosuppression. It is believed that spontaneous *C. septicum* infections may follow an opportunistic mechanism. This belief finds support in the fact that the mortality after postoperative or posttraumatic *C. septicum* infections is rather low [13]. It is moreover theorized that acidic environment and anaerobic glycolysis within active tumors may be conductive to spore germination [12, 18, 19]. Our patient had been an insulin-dependent type II diabetic for many years. Furthermore, he suffered from a locally advanced long distance colonic cancer, as the postmortem examination showed. He therefore had two risk factors to develop a spontaneous gas gangrene with *Clostridium septicum*. 

We present a case of a completely documented rare infectious disease with triage findings including initial vital signs, initial medical findings, the emergency lab, radiological, intraoperative, histopathological, microbiological, and postmortem results. We hope that this documentation will help health care providers to identify this life-threatening disease as early as possible in future cases. 

## Figures and Tables

**Table 1:** Emergency laboratory results (selection of parameters of interest).

Parameter	Value
Hemogram	
RBC	11.3 g/dL
Leucocytes	9.5 Mrd/L
Thrombocytes	178 Mrd/L
Clinical chemistry	
C-Reactive protein	96 mg/L
Procalcitonin	18.67 *µ*g/L
Creatinine	3.10 mg/dL
Urea	29 mg/dL
GOT	392 U/L
GPT	80 U/L
Creatin kinase	19216 U/L
Coagulation	
Quick (INR)	20% (2.83)
Blood gas analysis	
pH	7.10
Bicarbonate	10.8 mmol/L
Base excess	−17.4 mmol/L
Lactate	10.0 mmol/L
Bilirubin	2.1 mg/dL
